# A Text Book on the Diseases of the Eye

**Published:** 1890-09

**Authors:** 


A Text Book on the Diseases of the Eye. By Henry D. Noyes, A. M., M. D., Professor of Ophthalmology and Otology in Bellevue Hospital Medical College; Executive Surgeon to the New York Eye and Ear Infirmary; recently President of the American Ophthalmological Society, etc., etc. Royal octavo, 733 pages, richly illustrated with chromo-lithographic plates and 236 engravings. Price, bound in extra muslin, $6.00 ; in sheep, $7.00. New York: William Wood & Company.

We have reviewed the pages of the above work with that keen appreciation for anything written or said by Henry D. Noyes.

His proven skill needs no other eulogy than acquaintance with the record of his achievements ; and for it he is entitled to speak with authority on this most delicate and difficult of specialties—the eye.

The work exhibits a carefulness and elaborateness in the organization and arrangement of its plainly told truths worthy the gigantic undertaking.

The knowledge displayed is the result of long and close investigation under favorable conditions by a mind practice-trained and naturally inquiring and incisive.

Equally adapted to the beginner for its per-specimen definitions, and to the veteran specialist for its vast amount of detailed experience it cannot be long to assume the post of authority which its merit deserves.

Splendidly illustrated, with clear cut representations of healthy and morbid anatomy of the organ, viewed from different standpoints and under varying classes of eye affections, each adorned page affords us pleasure and instruction. W. S. K.

